# Erratum: An innate defense peptide BPIFA1/SPLUNC1 restricts influenza A virus infection

**DOI:** 10.1038/mi.2017.109

**Published:** 2018-05-30

**Authors:** K M Akram, N A Moyo, G H Leeming, L Bingle, S Jasim, S Hussain, A Schorlemmer, A Kipar, P Digard, R A Tripp, R V Shohet, C D Bingle, J P Stewart

**Correction to:** *Mucosal Immunology* (2017); advance online publication, 17 May 2017; doi:10.1038/mi.2017.45

This article was originally published under NPG's License to Publish, but has now been made available under a [CC BY 4.0] license. The PDF and HTML versions of the paper have been modified accordingly.

